# Weberviruses are gut-associated phages that infect *Klebsiella* spp

**DOI:** 10.1093/femsec/fiaf043

**Published:** 2025-04-18

**Authors:** Samuel J T Dawson, Preetha Shibu, Sara Garnett, Fiona Newberry, Thomas C Brook, Tobi Tijani, Magdalena Kujawska, Lindsay J Hall, Anne L McCartney, David Negus, Lesley Hoyles

**Affiliations:** Department of Biosciences, Nottingham Trent University, Nottingham, NG11 8NS, United Kingdom; Life Sciences, University of Westminster, London, W1W 6UW, United Kingdom; Department of Biosciences, Nottingham Trent University, Nottingham, NG11 8NS, United Kingdom; Department of Biosciences, Nottingham Trent University, Nottingham, NG11 8NS, United Kingdom; Life Sciences, University of Westminster, London, W1W 6UW, United Kingdom; Life Sciences, University of Westminster, London, W1W 6UW, United Kingdom; Intestinal Microbiome, ZIEL—Institute for Food & Health, Technical University of Munich, Freising 85354, Germany; Microbes, Infection & Microbiomes, College of Medicine & Health, University of Birmingham, Birmingham, B15 2TT, United Kingdom; Institute of Microbiology and Infection, University of Birmingham, Birmingham, B15 2TT, United Kingdom; Intestinal Microbiome, ZIEL—Institute for Food & Health, Technical University of Munich, Freising 85354, Germany; Microbes, Infection & Microbiomes, College of Medicine & Health, University of Birmingham, Birmingham, B15 2TT, United Kingdom; Institute of Microbiology and Infection, University of Birmingham, Birmingham, B15 2TT, United Kingdom; Gut Microbes & Health, Quadram Institute Bioscience, Norwich Research Park, Norwich, NR4 7UQ, United Kingdom; Norwich Medical School, University of East Anglia, Norwich Research Park, Norwich, NR4 7TJ, United Kingdom; Department of Biosciences, Nottingham Trent University, Nottingham, NG11 8NS, United Kingdom; Department of Biosciences, Nottingham Trent University, Nottingham, NG11 8NS, United Kingdom; Department of Biosciences, Nottingham Trent University, Nottingham, NG11 8NS, United Kingdom

**Keywords:** microbiota, comparative genomics, environment, metagenome-assembled genomes, *Klebsiella pneumoniae*

## Abstract

Weberviruses are bacteriophages (phages) that can infect and lyse clinically relevant, multidrug-resistant (MDR) strains of *Klebsiella*. They are an attractive therapeutic option to tackle *Klebsiella* infections due to their high burst sizes, long shelf life, and associated depolymerases. In this study, we isolated and characterized seven new lytic phages and compared their genomes with those of their closest relatives. Gene-sharing network, ViPTree proteome, and *terL* gene-sequence-based analyses incorporating all publicly available webervirus genomes [*n* = 258 from isolates, *n* = 65 from metagenome-assembled genome (MAG) datasets] confirmed the seven phages as members of the genus *Webervirus* and identified a novel genus (*Defiantjazzvirus*) within the family *Drexlerviridae*. Using our curated database of 265 isolated phage genomes and 65 MAGs (*n* = 330 total), we found that weberviruses are distributed globally and primarily associated with samples originating from the gut: sewage (154/330, 47%), wastewater (83/330, 25%), and human faeces (66/330, 20%). We identified three distinct clusters of potential depolymerases encoded within the 330 genomes. Due to their global distribution, frequency of isolation and lytic activity against the MDR clinical *Klebsiella* strains used in this study, we conclude that weberviruses and their depolymerases show promise for development as therapeutic agents against *Klebsiella* spp.

## Teaser

Combining data from isolated phages and metagenome-assembled genomes greatly expands the availability of curated phage genome sequences for use in genomic and ecological studies.

## Introduction

Members of the *Klebsiella pneumoniae* species complex are opportunistic pathogens that can cause serious hospital-acquired infections and are major contributors to global deaths associated with antimicrobial resistance (Antimicrobial Resistance Collaborators 2022). Carbapenem-resistant isolates of *K. pneumoniae* are resistant to a range of frontline β-lactam antibiotics (Antimicrobial Resistance Collaborators 2022, Tsang et al. 2024). The difficulty of treating infections caused by such isolates with conventional antibiotics has resulted in the investigation of new therapeutic modalities, including bacteriophages (phages; viruses that infect and often kill bacteria) and their gene products (Herridge et al. 2020). To realize the potential of phage therapy, it is important to comprehensively characterize phages with clinical potential. Previously we isolated *Webervirus KLPN1* from the caecum of a healthy female, along with its host *K. pneumoniae* subsp. *pneumoniae* L4-FAA5 (Hoyles et al. 2015). In the current study, we successfully identified seven new representatives of the genus *Webervirus* using L4-FAA5 and multidrug-resistant (MDR) clinical isolates of *Klebsiella* spp. as isolation hosts. These hosts included *K. pneumoniae* PS_misc6, which encodes the carbapenem-degrading metallo-β-lactamase NDM, and *Klebsiella variicola* PS_misc5, a carbapenem-resistant clinical isolate that encodes the class D β-lactamase OXA-48 (Shibu 2019).

As of 19 January 2025, the genus *Webervirus* encompassed 100 different phage species [International Committee on Taxonomy of Viruses (ICTV)]. With the exception of *Webervirus BUCT705* (isolated on *Stenotrophomonas maltophila*), all weberviruses described to date have been isolated on *Klebsiella* hosts, and have proven easy to recover from sewage, wastewater and, occasionally, intestinal contents (Herridge et al. 2020). Although *Webervirus F20* was originally described as being isolated on *Enterobacter aerogenes* (Mishra et al. 2012), this bacterium has subsequently been reclassified as *Klebsiella aerogenes*. Their high burst sizes (∼80 pfu/cell with a reported range between 27 and 142 pfu/cell) (Fang et al. 2022, Gilcrease et al. 2023, Zurabov and Zhilenkov 2021, Li et al. 2024, Senhaji-Kacha et al. 2024, Ziller et al. 2024) and long shelf life make weberviruses ideal phages to work with for biotechnological and clinical applications (Herridge et al. 2020, Fang et al. 2022).

Currently, more than 130 different capsule types (K types) have been identified for *K. pneumoniae* by genetic analysis (Follador et al. 2016). Specific *K. pneumoniae* capsule types are strongly associated with virulence. For example, hypervirulent *K. pneumoniae* isolates are typically associated with capsule types K1, K2, K16, K28, K57, and K63 (Mizuta et al. 1983, Kabha et al. 1995, Yu et al. 2008, Lee et al. 2016, Marr and Russo 2019). Additionally, capsule production by *Klebsiella* spp. has been implicated in protection from complement-mediated lysis and is recognized to play an important role in biofilm formation (Alvarez et al. 2000, Jensen et al. 2020). Capsule type has also been shown to be a major determinant of host tropism in *Klebsiella* phages (Beamud et al. 2023). Bacterial capsules are known to prevent phage attachment by masking cell-surface-associated receptor proteins (Scholl et al. 2005, Dunstan et al. 2021). To overcome this physical barrier, phages encode enzymes—frequently referred to as depolymerases—that selectively degrade polysaccharides or polypeptides that comprise the bacterial capsule (Hoyles et al. 2015, Majkowska-Skrobek et al. 2016, Dunstan et al. 2021, Pertics et al. 2021, Cai et al. 2023). Weberviruses tend to have narrow host ranges (Hoyles et al. 2015, Pertics et al. 2021). However, our previous (and ongoing) work has suggested that their depolymerases can degrade the capsules of non-host *Klebsiella* spp. (Hoyles et al. 2015). Depolymerase activity is common to weberviruses, and is being actively investigated as a tool to hydrolyse capsules of *Klebsiella* spp. that often hinder or make treatment with antimicrobials difficult (Majkowska-Skrobek et al. 2016, Cai et al. 2019, Pertics et al. 2021). For example, the webervirus depolymerase Depo32 has been shown to protect mice from otherwise lethal *K. pneumoniae* infections in a mouse model of disease (Cai et al. 2023). In addition, a webervirus (P39) has recently been used in combination with another lytic phage (P24, *Przondovirus*) to decolonize mice of carbapenem-resistant *K. pneumoniae* (Fang et al. 2022).

Here, we describe our new webervirus phages and their lytic and depolymerase activities against clinical MDR *Klebsiella* spp., and compare their genomes with those of their closest relatives. The increased ease with which metagenome-associated viruses can be interrogated via PhageClouds (Rangel-Pineros et al. 2021) and NCBI also led us to determine whether weberviruses are readily detectable within recent metagenome-derived phage datasets.

## Methods

### Bacterial strains

Details of all *Klebsiella* strains included in this study are given in Table 1. The antimicrobial resistance profiles of the isolates, determined according to EUCAST guidelines as described previously (Shibu et al. 2021), can be found in Supplementary Table 1.

**Table 1.** Strains of *Klebsiella* included in this study and their phage infection profiles.

				Infection type^d^							
Strain^a^	Source	K:O type^b^	MLST^c^	KLPN1	KLPN2	KLPN3	KLPN4	KLPN5	KLPN6	KLPN7	KLPN8
L4-FAA5	Human caecum (Hoyles et al. 2015)	K2:O1ab	ST380	++++ d	++++ d	++++ d	++++ d	0	0	0	0
PS_misc2	Groin (Shibu 2019)	K64:O1ab	ST14	0	0	0	0	0	0	0	0
PS_misc3	Rectum (Shibu 2019)	K52:O13	ST45	0	0	0	0	0	0	0	0
PS_misc5	Rectum (Shibu 2019)	K81:O13	ST1737-1LV	0	0	0	0	++++ d	++++ d	++++ d	0
PS_misc6	Rectum (Shibu 2019)	U:O2a	ST716	0	0	0	0	0	0	0	+++ d
PS_misc7	Rectum (Shibu 2019)	K30:O1ab	ST294	0	0	0	0	0	0	0	0
PS_misc8	Perineum (Shibu 2019)	K52:O13	ST45	0	0	0	0	0	0	0	0
PS_Kpn1	Perineum (Shibu 2019)	K52:O13	ST14-1LV	+++	++	++	++	0	0	0	0
PS_Kpn2	Rectum (Shibu 2019)	K64:O1ab	ST14	+++	+++	+++	++	0	0	0	0
PS_Kpn3	Rectum (Shibu 2019)	K64:O3b	ST15-1LV	0	0	0	0	0	0	0	0
PS_Kpn4	Cross-infection (Shibu 2019)	K64:O1ab	ST15	0	0	0	0	0	0	0	0
PS_Kpn7	Rectum (Shibu 2019)	K2:O1ab	ST14	++	++	++	++	0	0	0	0
PS_Kpn9	Rectum (Shibu 2019)	K18:O2a	ST515-1LV	0	0	0	0	0	0	0	0
PS_Kpn10	Rectum (Shibu 2019)	K2:O1ab	ST14	++++	+++	+++	++	0	0	0	0
PS_Kpn11	Urine (Shibu 2019)	U:O2afg	ST258	0	0	0	0	0	0	0	0
PS_Kpn12	Rectum (Shibu 2019)	K15:O4	ST11	0	0	0	0	0	0	0	0
PS_Kpn13	Rectum (Shibu 2019)	K2:O1ab	ST14	+++	+++	+++	0	0	0	0	0
PS_Kpn14	Rectum (Shibu 2019)	K2:O1ab	ST14	++++	+++	+++	++	0	0	0	0
PS_Kpn15	Rectum (Shibu 2019)	K52:O13	ST45	0	0	0	0	0	0	0	0
PS_Kpn16	Rectum (Shibu 2019)	K17:O1ab	ST101	0	0	0	0	0	0	0	0
PS_Kpn24	Rectum (Shibu 2019)	K2:O1ab	ST14	++	++	++	++	++	++	++	0
PS_Kpn25	Rectum (Shibu 2019)	K2:O1ab	ST14	+++	++	++	++	0	0	0	0
PS_Kpn26	Rectum (Shibu 2019)	K64:O2a	ST147	0	0	0	0	0	0	0	0
PS_Kpn27	Urine (Shibu 2019)	U:O2afg	ST258	0	0	0	0	0	0	0	0
PS_Kpn28	Urine (Shibu 2019)	K2:O1ab	ST14	++++	+++	++	++	0	0	0	0
PS_Kpn29	Urine (Shibu 2019)	K2:O1ab	ST14	++	++	++	++	0	0	0	0
PS_Kpn30	Mouth (Shibu 2019)	U:O1ab	ST15	0	0	0	0	0	0	0	0
PS_Kpn31	Perineum (Shibu 2019)	K2:O1ab	ST14	++++	++	++	++	0	0	0	0
PS_Kpn32	Drain fluid (Shibu 2019)	K22:O1ab	ST35	0	0	0	0	0	0	0	0
PS_Kpn33	Urine (Shibu 2019)	K2:O1ab	ST14	+++ (d)	+++ (d)	++	++	0	0	0	0
PS_Kpn35	Urine (Shibu 2019)	K2:O1ab	ST14	+++ (d)	+++ (d)	++	++	0	0	0	0
PS_Kpn36	Urine (Shibu 2019)	K24:O2a	ST11	0	0	0	0	0	0	0	0
PS_Kpn37	Wound (Shibu 2019)	K51:O3b	ST16	0	0	0	0	0	0	0	0
PS_Kpn38	High vaginal swab (Shibu 2019)	K2:O1ab	ST14	+++	++	+++	++	0	0	0	0
PS_Kpn39	Wound (Shibu 2019)	K64:O1ab	ST14	0	0	0	0	0	0	0	0
PS_Kpn40	Wound (Shibu 2019)	U:O2afg	ST512	0	0	0	0	0	0	0	0
PS_Kpn41	Leg (Shibu 2019)	U:O1ab	ST15	0	0	0	0	0	0	0	0

a All strains with PS prefix identified as *K. pneumoniae* subsp. *pneumoniae* by average nucleotide identity and phylogenetic analyses against type strains of the genus *Klebsiella*, except for PS_misc5 (*K. variicola* subsp. *variicola*) (Shibu 2019).

b Determined using Kleborate. U, untypeable. Full Kleborate dataset available in Supplementary Table 1.

c MLST, multi-locus sequence type determined using Kleborate.

d The appearance of the spot was graded according to Haines et al. (2021): ++++, complete lysis; +++, lysis with resistant colonies; ++, hazy lysis; +, visible plaques; 0, no visible plaques. We also noted whether depolymerase activity was detected (i.e. formation of haloes around plaques): d, depolymerase activity; (d), weak depolymerase activity detected.

### Generation of sequence data for bacterial isolates

Genomes for clinical strains included in this study were generated as described previously (Shibu et al. 2021). Illumina and Oxford Nanopore Technologies sequence data for *K. pneumoniae* L4-FAA5 were generated by microbesNG (Birmingham, UK) as described previously (Newberry et al. 2023). CheckM2 v0.1.3 (Chklovski et al. 2022) was used to confirm the quality (in terms of completeness and contamination) of all assembled genomes. Kleborate v3.1.2 (Wyres et al. 2016, Lam et al. 2021) was used to assign sequence types (STs), and capsule (K) and lipopolysaccharide (O) types to genomes.

### Isolation and propagation of phages

Filter-sterilized sewage samples were screened for phages as described previously (Smith-Zaitlik et al. 2022) using *Klebsiella* strain L4-FAA5, PS_misc5 or PS_misc6 as inoculum (Table 1). Pure phage stocks were prepared from phage-positive samples as described previously (Hoyles et al. 2015).

### Isolation of phage DNA

Phages vB_KpnS-KLPN2, vB_KpnS-KLPN3, and vB_KpnS-KLPN4 were precipitated from 100 ml of each lysate as described previously (Hoyles et al. 2015). Phages vB_KvaS-KLPN5, vB_KvaS-KLPN6, vB_KvaS-KLPN7 and vB_KpnS-KLPN8 were concentrated and DNA extracted as described previously (Smith-Zaitlik et al. 2022).

### Transmission electron microscopy

Transmission electron micrographs (TEMs) of phages vB_KpnS-KLPN2, vB_KpnS-KLPN3, and vB_KpnS-KLPN4 were generated as described previously (Hoyles et al. 2015). TEMs for phages vB_KvaS-KLPN5, vB_KvaS-KLPN6, vB_KvaS-KLPN7, and vB_KpnS-KLPN8 were generated and analysed as described previously (Smith-Zaitlik et al. 2022).

### Phage genome sequencing, assembly, and annotation

Assembled genomes (from Illumina short-read sequences) for phages vB_KpnS-KLPN2, vB_KpnS-KLPN3, and vB_KpnS-KLPN4 were generated by microbesNG (Shibu et al. 2021). For phages vB_KvaS-KLPN5, vB_KvaS-KLPN6, vB_KvaS-KLPN7, and vB_KpnS-KLPN8, sequence data were generated on an Illumina MiSeq at Nottingham Trent University (Smith-Zaitlik et al. 2022). Quality of raw sequence data was assessed using FastQC v0.11.9. Reads had a mean phred score above 30 and no adapter contamination, so data were not trimmed.

All genomes were assembled using SPAdes v3.13.0 (default settings) (Bankevich et al. 2012), and visualized to confirm circularization of genomes using Bandage v0.8.1 (Wick et al. 2015). CheckV v1.0.1 (checkv-db-v1.5; Nayfach et al. 2021a) was used to determine contamination and completeness of the genomes. Genes in all phage genomes included in this study (Supplementary Table 2) were predicted and annotated using Pharokka v1.6.1 (v1.4.0 databases) (Bouras et al. 2023).

### Comparison of webervirus genomes

ViPTree v4.0 (Nishimura et al. 2017) was used to determine whether the seven phage genomes were closely related to previously described double-stranded DNA viruses. Based on our initial findings (not shown), we curated a list of all known webervirus sequences available from NCBI GenBank on 19 January 2025. We also identified unclassified weberviruses and closely related phage in NCBI using the INPHARED database (1 January 2025 dataset; Cook et al. 2021) and vConTACT v2.0 (Supplementary Table 2).

### Identification of weberviruses in metagenomic datasets

We used PhageClouds (Rangel-Pineros et al. 2021) to identify relatives of weberviruses in metagenome-assembled genome (MAG) datasets. PhageClouds is an online resource that allows researchers to search a reference dataset of ∼640 000 phage genomes for phages with genomes related to query sequences. The genome of *Webervirus KLPN1* was searched against the PhageClouds database with a threshold of 0.15, as we had previously looked for relatives of this phage in metagenomic datasets and are interested in gut-associated phage communities (Hoyles et al. 2015). The nucleotide sequences of the relevant phage MAGs (from Gregory et al. 2020, Camarillo-Guerrero et al. 2021, Tisza and Buck 2021) were recovered from the relevant datasets.

Additionally, webervirus MAGs were identified using a search of the NCBI nucleotide database for *Bacteriophage* sp. [search term: (txid38018) AND MAG]; the sequences (*n* = 8138) were filtered for genomes of between 30 Kbp and 60 Kbp in length (*n* = 2540): genes were predicted using Prodigal v.2.6.3 (Hyatt et al. 2010), and the proteomes added to the INPHARED database and analysed using vCONTact2. CheckV was used (as described above) to determine contamination and completeness of the MAG dataset (Supplementary Table 3).

The MAG sequences were analysed using ViPTree v4.0 to confirm their affiliation with the genus *Webervirus*. They were also annotated with Pharokka and included in a vConTACT2 analysis with our curated set of webervirus genomes. The genomes of all weberviruses were compared with one another using taxmyPHAGE v0.3.3, which uses a Python implementation of the VIRIDIC algorithm to calculate intergenomic genomic similarities (Millard et al. 2024). The matrix created from the similarity values was visualized using tidyheatmaps v0.2.1 (Mangiola and Papenfuss 2020).

### Phylogenetic relationships among weberviruses

Nucleotide sequences of the large-subunit terminase (*terL*) genes, predicted by Pharokka, were used to create a multiple-sequence alignment (Clustal Omega 1.2.2 implemented in Geneious Prime v2024.0.5; options—group sequences by similarity, 5 representative iterations). This alignment was used to create a bootstrapped (100 replicates) maximum-likelihood tree (PhyML v3.3.20180621, JC69 algorithm).

### Distribution of weberviruses

The distribution of weberviruses was determined by identifying the source and geographical location information for the GenBank genomes (including our seven new genomes; Supplementary Table 2) and the MAGs (Supplementary Table 3). Data were aggregated based on isolation source or geographical location, with these latter data visualized using the R package rworldmap v1.3.8 (South 2011).

### Host prediction for MAGs

The CRISPR Spacer Database and Exploration Tool (Dion et al. 2021) and HostPhinder 1.1 (Villarroel et al. 2016) were used to predict hosts for the weberviruses recovered from metagenomic datasets. MAGs were also subject to a BLASTN search against the Unified Human Gastrointestinal Genome (UHGG) CRISPR spacer database according to Nayfach et al. (2021b). For this, a BLASTN database was created from 1846 441 spacers from 145 053 CRISPR arrays from 79 735 UHGG genomes (Nayfach et al. 2021b). Spacers were searched against viral genomes using BLASTN from the blast+ package v.2.12.0 (options: -dust=no; -word-size=18); a maximum of one mismatch or gap was allowed over ≥95% of the spacer length. iPHoP v1.3.3 (Roux et al. 2023), an automated command-line pipeline for predicting host genus of novel bacteriophages and archaeoviruses based on their genome sequences, was also used to analyse the MAGs.

### Identification of potential depolymerases among weberviruses

A BLASTP database was created using amino acid sequences from experimentally validated webervirus depolymerases, and a BLASTP search was run versus all webervirus genomes. Sequences used to build the BLAST database are available from figshare (doi:10.6084/m9.figshare.28603070). Clustal Omega v1.2.2 alignments were created in Geneious Prime (default settings; 2024.0.5). RAxML v 8.2.11 (-m PROTGAMMABLOSUM62 -f a -x 1 -N 100 -p 1) was used to generate a bootstrapped (100 replicates) maximum-likelihood tree from the multiple-sequence alignment.

## Results

### Seven new weberviruses lyse a range of clinically relevant *Klebsiella* spp.

Seven phages were isolated on two different strains of *K. pneumoniae* subsp. *pneumoniae* (L4-FAA5–vB_KpnS-KLPN2, vB_KpnS-KLPN3, vB_KpnS-KLPN4; PS_misc6–vB_KpnS-KLPN8) and one strain of *K. variicola* subsp. *variicola* (PS_misc5–vB_KvaS-KLPN5, vB_KvaS-KLPN6, vB_KvaS-KLPN7). All our sewage samples yielded *Klebsiella*-infecting phages. Strain L4-FAA5 (K2: O1ab, ST380) was originally isolated from human caecal effluent along with *Webervirus KLPN1* (Hoyles et al. 2015), while strains PS_misc5 (K81: O13, ST1737-1LV) and PS_misc6 (untypeable: O2a, ST716) were part of a collection (*n* = 36) of clinical MDR and/or carbapenem-resistant *Klebsiella* isolates currently being used in our laboratory in phage-related and other studies (Shibu 2019) (Supplementary Table 1).

TEM showed the seven phages had a mean capsid diameter of 57.5 nm and a mean tail length of 157.5 nm (Supplementary Fig. A ). Host-range analysis showed the seven phages had different infection profiles (Table 1). KLPN1, our original webervirus isolated on *K. pneumoniae* L4-FAA5 (Hoyles et al. 2015), was included in analyses for comparative purposes. KLPN1, vB_KpnS-KLPN2, vB_KpnS-KLPN3, and vB_KpnS-KLPN4 completely lysed some, but not all, clinical isolates of *K. pneumoniae* with capsule: O antigen types K52: O13 and K64: O1ab. K2: O1ab isolates alone were infected by KLPN1 and vB_KpnS-KLPN2 to vB_KpnS-KLPN4, though vB_KpnS-KLPN4 was unable to infect one of the K2: O1ab strains (PS_Kpn13). Only on strain L4-FAA5 (K2: O1ab), isolated from human caecal effluent, was strong depolymerase activity observed with phages KLPN1 and vB_KpnS-KLPN2. Hazy lysis of strain PS_Kpn24 (K2: O1ab) was observed with phages vB_KvaS-KLPN5, vB_KvaS-KLPN6 and vB_KvaS-KLPN7. Phages vB_KvaS-KLPN5 to vB_KvaS-KLPN7 showed strong lytic and depolymerase activity on *K. variicola* PS_misc5 (K81: O13) alone, while vB_KpnS-KLPN8 lysed *K. pneumoniae* PS_misc6 (untypeable capsule: O2a) with depolymerase activity on this host.

### Genome-based analyses of publicly available sequence data triples the number of authenticated webervirus genomes

Bandage (data not shown) and CheckV (Supplementary Table 2) analyses confirmed the genomes of vB_KpnS-KLPN2, vB_KpnS-KLPN3, vB_KpnS-KLPN4, vB_KvaS-KLPN5, vB_KvaS-KLPN6, vB_KvaS-KLPN7 and vB_KpnS-KLPN8 were circular and complete. None of the genomes was contaminated. An initial online ViPTree analysis showed vB_KpnS-KLPN2, vB_KpnS-KLPN3, vB_KpnS-KLPN4, vB_KvaS-KLPN5, vB_KvaS-KLPN6, vB_KvaS-KLPN7, and vB_KpnS-KLPN8 belonged to the genus *Webervirus* (data not shown). All publicly available webervirus genomes (available as of 19 January 2025) were downloaded from GenBank to allow comparison with our newly sequenced phages and for inclusion in the INPHARED vCONTact2 database if not already included in the 1 January 2025 release. Among the other 264 genomes from phage isolates included in this study, 226 were of high quality, 43 were complete and two were of medium quality; none of these genomes was contaminated.

In addition, we used PhageClouds to identify potential webervirus MAGs. Fifty-four of the PhageClouds hits represented MAGs derived from the Gut Phage Database (GPD) (Camarillo-Guerrero et al. 2021), six were from the Cenote Human Virome Database (CHVD) (Tisza and Buck 2021), and two were from the Gut Virome Database (GVD) (Gregory et al. 2020). MAG Ma_2019_SRR413710_NODE_378_length_50 715_cov_48.086538 from the GVD was identical to uvig_330 395 from the GPD (Camarillo-Guerrero et al. 2021) so was removed from further analyses [PhageClouds scores identical, 100% pairwise identity as assessed using VIRIDIC; an unsurprising finding as both MAGs are derived from the same dataset (Ma et al. 2018)]. Similarly, two MAGs from the GPD were also found to be identical: uvig_314 355 and uvig_315 584 were high-quality genomes both derived from the same four samples [SRR1952259, SRR1162648, SRR1162662, SRR1162654 (Tisza and Buck 2021)]; only uvig_314 355 was retained for further analyses. Our inclusion of NCBI genomes listed as Bacteriophage sp. in a vCONTact2 analysis with the INPHARED database identified a further five potential webervirus MAGs recovered from faecal samples in Japan (Nishijima et al. 2022). In total, our dataset included 65 MAGs. The MAGs ranged from 10 230 to 55 276 nt (mean 42 392 nt) in length (Supplementary Table 3). Forty-seven of the 65 MAGs were determined to be complete or of high-quality (CheckV). Eight were of medium-quality and 10 were low quality, representing genome fragments (Supplementary Table 3). None of the MAGs was contaminated.

**Figure 1.**
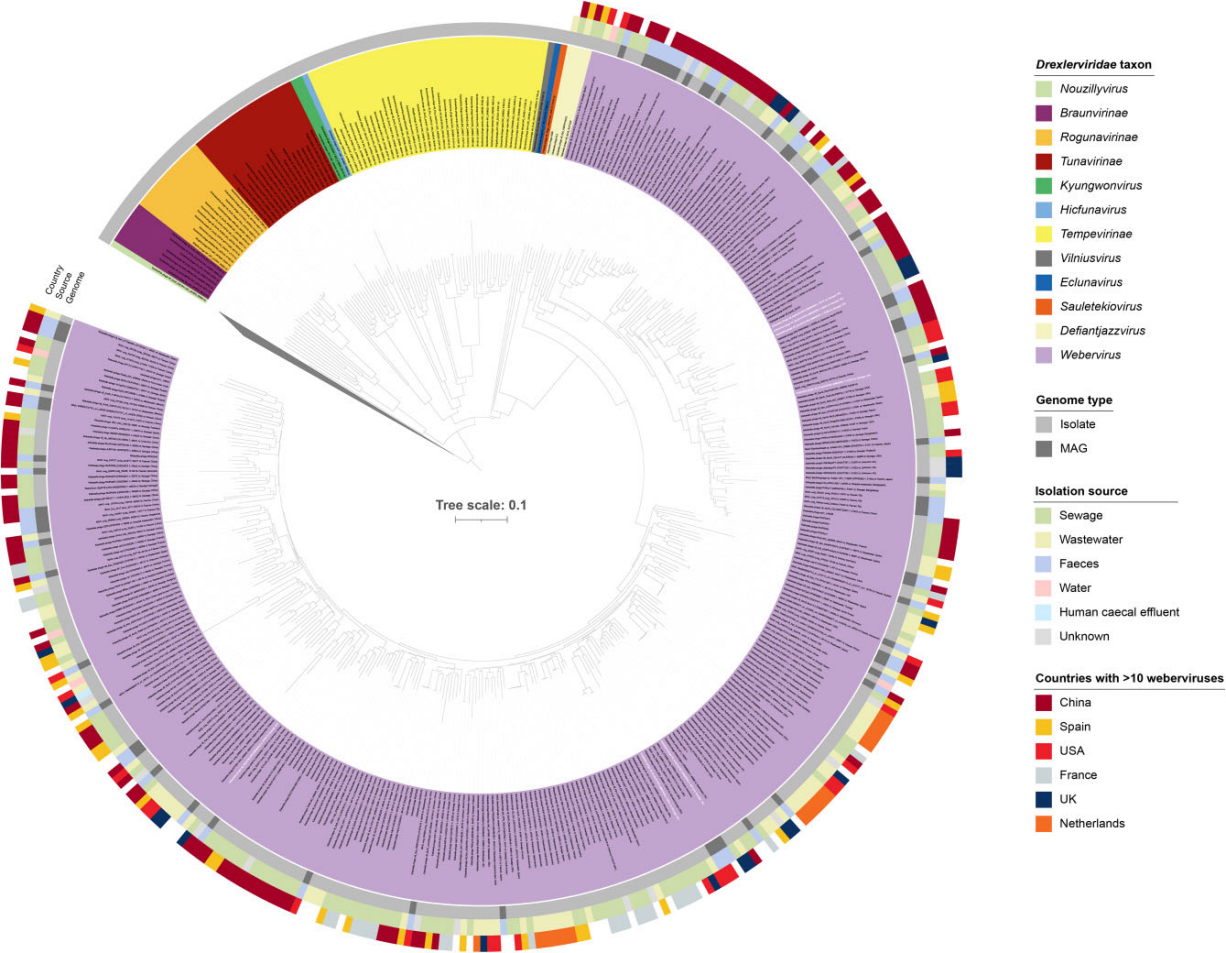
ViPTree-generated phylogenetic analysis of the family *Drexlerviridae*. The genus *Webervirus* is represented by 330 genomes. The names of our seven newly identified weberviruses are shown in white bold text. A potentially novel genus (*Defiantjazzvirus*) was identified during the curation of our dataset. The colours covering the virus names represent taxa within the family *Drexlerviridae*; the outgroup has been collapsed to aid visualization. The tree (ViPTree bionj) was rooted at the midpoint.

In addition to the 100 recognized weberviruses included in the ICTV and our seven new weberviruses, we identified 158 more webervirus genomes in NCBI and 65 webervirus MAGs. The 265 weberviruses isolated on bacteria mostly infected *K. pneumoniae* (Supplementary Fig. B). A ViPTree analysis confirmed the affiliation of our 330 genomes with the genus *Webervirus* (Fig. 1). The webervirus genomes often clustered based on geographical origin, irrespective of whether they came from phage isolates or MAGs (Fig. 1).

**Figure 2.**
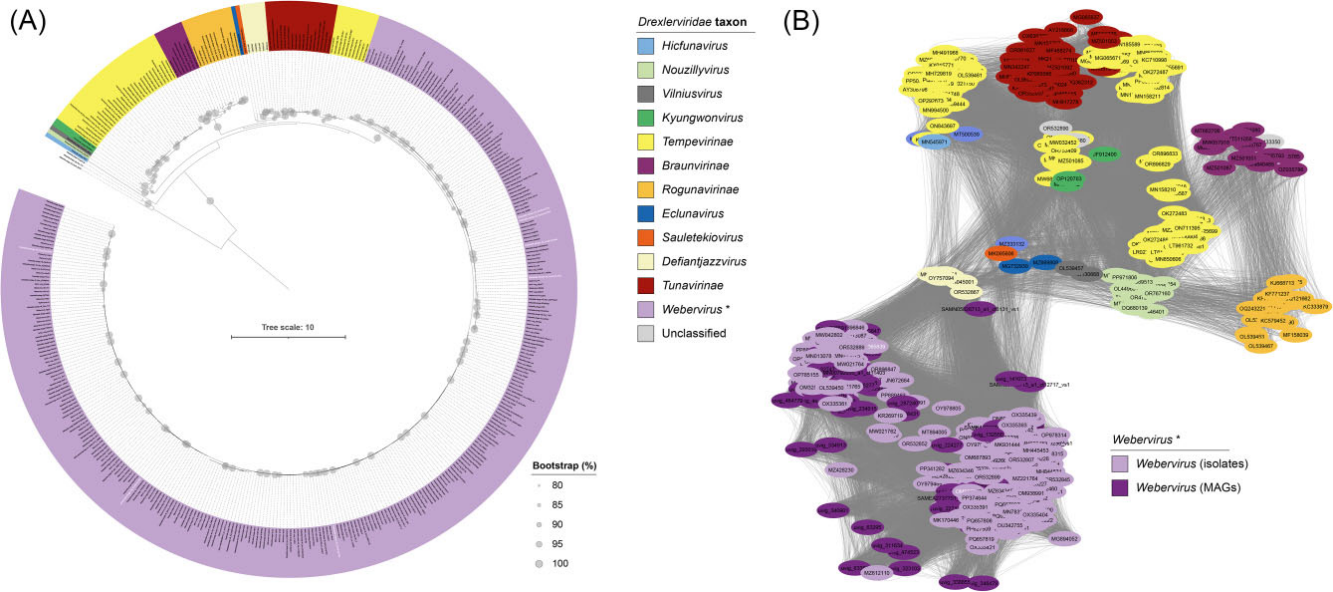
Further analyses of *Drexlerviridae* sequence data. (A) Phylogenetic relationships (maximum-likelihood tree) of members of the family *Drexlerviridae* based on analysis of large-subunit terminase (*terL*) nucleotide sequences encoded in phage genomes. Bootstrap values are expressed as a percentage of 100 replications; scale bar, mean number of nucleotide substitutions per position; the tree is rooted at the midpoint. (B) Gene-network-based analysis of proteomic data for members of the genus *Webervirus* and their nearest relatives. Full network shown in Supplementary Fig. C. (A and B) The legend shown applies to both figures, with isolate and MAG proteomes differentiated in (B). Names of our seven newly identified weberviruses are shown in bold white text.

The monophyletic nature of the genus *Webervirus* was confirmed by phylogenetic analysis of *terL* gene sequences (99% bootstrap support; Fig. 2A). A gene-sharing network was created with all webervirus genomes included in this study (Supplementary Table 2 and Supplementary Table 3) using vConTACT v2.0 (Bolduc et al. 2017, Bin Jang et al. 2019) and the INPHARED database (Supplementary Fig. C). The network was filtered based on first and second neighbours of webervirus proteomes (Supplementary Fig. C, Fig. 2B). The vConTACT-based analysis confirmed findings from the ViPTree- and *terL*-based analyses with respect to affiliation of weberviruses included in this study.

VIRIDIC analysis split the weberviruses into eight different clusters at the genus level, with most weberviruses affiliated with Cluster 1 (Supplementary Fig. D, Supplementary Table 4). Clusters 3 (uvig_338 855, uvig_63 295), 4 (uvig_346 479, uvig_474 523), 5 (SAMN05826713_a1_ct6131_vs1), 6 (uvig_63 387), 7 (uvig_340 901), 8 (uvig_334 913), and 9 (SAMN05826713_a1_ct12717_vs 1) were all associated with low-quality MAGs (Supplementary Table 3). MAGs in these clusters shared <70% identity with Cluster 1 phages (isolate and MAG genomes). The only other low-quality MAG included in the analysis (uvig_311 634) was affiliated with Cluster 1 phages, sharing 33%–72% identity with them and highest similarity with a MAG (uvig_141 073) in this cluster (Supplementary Table 4).

### Identification of a novel genus within the family *Drexlerviridae*

Our ViPTree analysis also identified a potential novel genus (referred to as *Defiantjazzvirus*) comprising six representatives within the family *Drexleviridae* and closely related to the genus *Webervirus* (Fig. 1). Analysis of *terL* gene sequences showed this genus to be monophyletic (97% bootstrap support; Fig. 2A). vConTACT-based analysis demonstrated that the six genomes associated with *Defiantjazzvirus* clustered together but separately from all other phage groups included in the analysis (Fig. 2B). VIRIDIC analysis showed defiantjazzvirus genomes to share 81%–97% genome identity with one another and 27%–42% identity with members of the genus *Webervirus* (Supplementary Fig. D, Supplementary Table 4). Based on current recommendations, the six genomes (sharing >70% nucleotide identity across their full-length genomes) represent a novel genus comprising five species (Supplementary Table 4) (Turner et al. 2021). Comparison of the defiantjazzvirus genomes with non-webervirus *Drexleviridae* genomes confirmed the genus *Defiantjazzvirus* represents a novel genus within the family *Drexleviridae*, with the six defiantjazzvirus genomes sharing between 0.2% and 35% genome identity with their closest non-webervirus relatives (Supplementary Table 5). Representatives of the genus *Defiantjazzvirus* infect *K. pneumoniae, K. michiganensis*, and *K. oxytoca* (Supplementary Table 2).

### Webervirus MAGs are predicted to infect *Klebsiella*

To confirm the MAGs were weberviruses that infected *Klebsiella* spp., we attempted to predict their bacterial hosts. CRISPR spacers can be used to predict hosts of unknown phages, as spacers represent biological records of past phage–bacteria interactions. Each of the seven new phage genomes (nt sequences) we generated was uploaded to CRISPR Spacer Database and Exploration Tool (Dion et al. 2021). None of the phages could be assigned to known hosts using this tool. Using the BLASTN approach of Nayfach et al. (2021b) with the MAG sequences, only SAMEA2737751_a1_ct5309 had sufficient coverage; this MAG had two hits to *Klebsiella* species (*K. pneumoniae* and *K. variicola*). iPHoP predicted hosts for 84/330 of the webervirus genomes included in this study; *Escherichia* was predicted to be the host for 21 of the MAGs and 59 of the isolates at the genome and genus levels (Supplementary Table 6). Only NC_049845.1, OR532813.1, OR532891, PQ337355, and PQ519586—all representing isolated phages (Supplementary Table 2)—were predicted to have a *Klebsiella* host at the genus level. HostPhinder 1.1 (Villarroel et al. 2016) was able to predict hosts for our KLPN phages, with all assigned to *Klebsiella pneumoniae*. Consequently, this tool was used to predict hosts for the *Webervirus* MAGs (Supplementary Table 7). All were predicted to infect *Klebsiella*.

### Depolymerases are readily detected in webervirus genomes

As our newly isolated phages all displayed apparent depolymerase activity against one or more hosts, we aimed to identify potential depolymerases encoded within the genomes of weberviruses. Detection and characterization of these enzymes may identify standalone therapeutics or help inform on host tropism. Currently, four experimentally validated depolymerases from weberviruses have been reported in the literature: depoKP36 (Majkowska-Skrobek et al. 2016), Depo32 (Cai et al. 2023), DpK2 (Dunstan et al. 2021), and B1dep (Pertics et al. 2021). These four depolymerases were used to create a BLASTP database to interrogate the 330 webervirus genomes for similar amino acid sequences.

Using thresholds of >50% coverage, >50% identity and sequence length >800 aa, 33/330 webervirus proteomes returned hits against the validated depolymerases (Fig. 4; Supplementary Table 8). Phylogenetic analysis and amino acid identity values revealed that the depolymerases clustered into three distinct groups, each with high bootstrap support (85%–100%; Fig. 4). Group 1 comprised four sequences and did not contain an experimentally validated depolymerase sequence. Group 2 contained four sequences, including the functionally characterized depolymerase depoKP36. Group 3 contained most of the sequences (26/33 predicted depolymerases) and included the characterized depolymerases DpK2, Depo32 and B1dep, and depolymerases encoded by four MAGs. Sequences belonging to Group 3 had a high level of conservation, as indicated by short branch lengths and sequence alignments (Supplementary Fig. E). Amino acid alignment of all 33 predicted depolymerases also revealed a high level of N-terminal sequence conservation.

## Discussion

Studies from a diverse range of geographical locations have reported the isolation or detection of weberviruses from samples associated with the human gut (e.g. wastewater, sewage, faeces, caecal effluent) (Herridge et al. 2020). To date, the majority of weberviruses have been isolated using *K. pneumoniae* as a host (Supplementary Fig. B). However, weberviruses have been reported to infect other *Klebsiella* spp., including *K. oxytoca* (Brown et al. 2017, Park et al. 2017) and *K. aerogenes* (Hudson et al. 2021). In the present study, we isolated seven new weberviruses from sewage samples, including three phages (vB_KvaS-KLPN5, vB_KvaS-KLPN6, vB_KvaS-KLPN7) that were isolated using a strain of *K. variicola* as the host (Fig. 1, Fig. 2, Supplementary  Fig. A). To our knowledge, this is the first report of weberviruses infecting *K. variicola*, a recognized emerging human pathogen (Rodríguez-Medina et al. 2019) increasingly associated with carbapenem and colistin resistance (Kim et al. 2023; Li et al. 2024).

As the majority of the *Klebisella* spp. sensitive to lysis by our webervirsues are MDR strains, the lytic phages isolated as part of this study represent attractive future therapeutics for the treatment of drug-resistant isolates belonging to the *K. pneumoniae* species complex.

In agreement with previous work (Hoyles et al. 2015, Pertics et al. 2021), the weberviruses described herein exhibited relatively narrow host ranges when screened against a panel of *Klebsiella* (including 36 clinical MDR) isolates representing a range of STs and capsule (K) types (Table 1). Phage host range is very much related to isolation host rather than phage phylogeny, with lysis appearing to be restricted based on K type. Phage-encoded depolymerases, therefore, contribute to host tropism, and previous studies have identified that weberviruses encode functionally active depolymerases (Majkowska-Skrobek et al. 2016, Dunstan et al. 2021, Pertics et al. 2021, Cai et al. 2023). While performing our host-range analysis, we observed the presence of haloes indicative of depolymerase activity for a small number of phage–host combinations and we, therefore, undertook a bioinformatic analysis (Fig. 4) to identify potential depolymerase enzymes encoded within webervirus genomes. Our BLASTP search identified 33 potential depolymerases which clustered into three distinct groups. The lack of an experimentally validated depolymerase sequence in Group 1 and the overall low amino acid identity shared with characterized webervirus depolymerases (<21%) make it difficult to draw conclusions related to the biological activity of these four proteins. Sequences OP978314.1_CDS_0059 and OP978315.1_CDS_0001 belong to a phage, and its evolved variant, respectively, which were characterized as part of the same study in Australia (Ngiam et al. 2024). These phages were propagated on *K. pneumoniae* 52 145 (K2: O1). According to NCBI, the isolation host of phage OP413832.1, which encodes predicted depolymerase OP413832.1_CDS_0043, is *K. pneumoniae* BS317-1 (K57: O1) (assembly accession GCF_015290145.1). No information is available for the isolation host of the phage OR532859.1, which encoded the remaining predicted Group 1 depolymerase. These data suggest that, if active, Group 1 depolymerases may hydrolyse K2 and/or K57 capsules. However, experimental validation is required.

Group 2 depolymerases are likely to be hydrolyse the K63 capsule as these sequences clustered with the experimentally validated depolymerase depoKP36, previously shown to degrade the K63 capsule of *K. pneumoniae* (Majkowska-Skrobek et al. 2016). Group 3 contained the majority of the predicted depolymerases, and all shared high sequence similarity with the webervirus depolymerases Depo32, DpK2, and B1dep (Supplementary Table 8). These enzymes have been shown to selectively degrade the *K. pneumoniae* K2 capsule (Dunstan et al. 2021, Pertics et al. 2021, Cai et al. 2023) and are highly likely to be specific for this capsule type. The high level of sequence identity observed at the N-terminal of all the identified depolymerases is likely due to this region being responsible for anchoring the baseplate of the phage virion, and as such it is often highly conserved (Knecht et al. 2019, Latka et al. 2019). Structural analysis of Depo32 from phage GH-K3 has revealed that, in addition to the N-terminal domain, Depo32 contains a short neck helix and connection domain (residues 186–271), a β-helix domain (residues 272–642), a connection helix domain (residues 643–666), a carbohydrate-binding module (residues 667–846), and a C-terminal domain (residues 847–907) (Cai et al. 2023). It is the β-helix domain that is responsible for hydrolysis of the polysaccharide capsule. Given the high level of amino acid identity between Depo32 and the amino acid sequences comprising Group 3, it is highly likely that these potential depolymerases are structurally similar.

We were unable to identify any coding sequences in the genomes of our isolated KLPN phages sharing high similarity to the four experimentally validated webervirus depolymerase sequences used to create our BLASTP database. Thus, it is likely that any depolymerase activity associated with the phages isolated in our study is due to enzyme(s) that remain to be characterized experimentally. As part of our previous analysis of the genome of phage KLPN1, we hypothesized that ORF34 and/or ORF35 may encode the depolymerase activity of phage KLPN1, as these sequences include a predicted endo-*N*-acetylneuraminidase/endosialidase domain (Hoyles et al. 2015). Further experimental work is required to determine whether these are functionally active depolymerases. As most of the plaques we observed had no discernible haloes, it may be that alternative mechanisms are used by weberviruses for penetrating the bacterial capsule. Depolymerase-independent penetration of the capsule by *Klebsiella* phages has been reported in the literature (Beamud et al. 2023).

A ViPTree proteome-based analysis of publicly available sequence data showed 330 genomes derived from isolated phages (*n* = 265) and MAGs (*n* = 65) belonged to the genus *Webervirus*, family *Drexlerviridae* (Fig. 1). Our gene-sharing network analysis supported this finding (Fig. 2B). Taxonomic assignment of phages using whole genome gene-sharing profiles has been shown to be highly accurate; a recent study showed that vConTACT2 produces near-identical replication of existing genus-level viral taxonomy assignments from the ICTV (Bin Jang et al. 2019). It has been suggested that genomes comprising a genus should be evaluated by phylogenetics with the use of ‘signature genes’ that are conserved throughout all members (Turner et al. 2021). Such analyses should always produce trees that are monophyletic. Using *terL* as a ‘signature gene’, we were able to show that the genus *Webervirus* is indeed monophyletic (Fig. 2A). To assess the number of different species present within the genus, we used VIRIDIC to determine the intergenomic similarity between phage genomes (Supplementary Fig. D; Supplementary Table 4). Guidelines suggest any two phages belong to the same species if they are more than 95% identical across their entire genome (Turner et al. 2021). Genus-level separation occurs when phage genomes share <70% nucleotide identity across their genome length (Turner et al. 2021). Based on these criteria, our results show that only weberviruses belonging to Cluster 1 represent species of *Webervirus sensu stricto*. Clusters 3–9, although identified as weberviruses using ViPTree and vConTACT2, do not represent species of *Webervirus*. The phage sequences associated with these clusters were derived from low-quality MAGs. As such, we recommend caution when using low-quality MAGs to determine taxonomic affiliations of *in silico*-generated phage sequences.

Cluster 2 phages were found to represent a novel genus (*Defiantjazzvirus*) of phage within the family *Drexlerviridae* (Figs 1 and 2, Supplementary Tables 4 and 5), with the genus *Defiantjazzvirus* most closely related to the genus *Webervirus*. All members of this novel genus reported to date infect a range of *Klebsiella* spp. (Supplementary Table 2).

**Figure 3.**
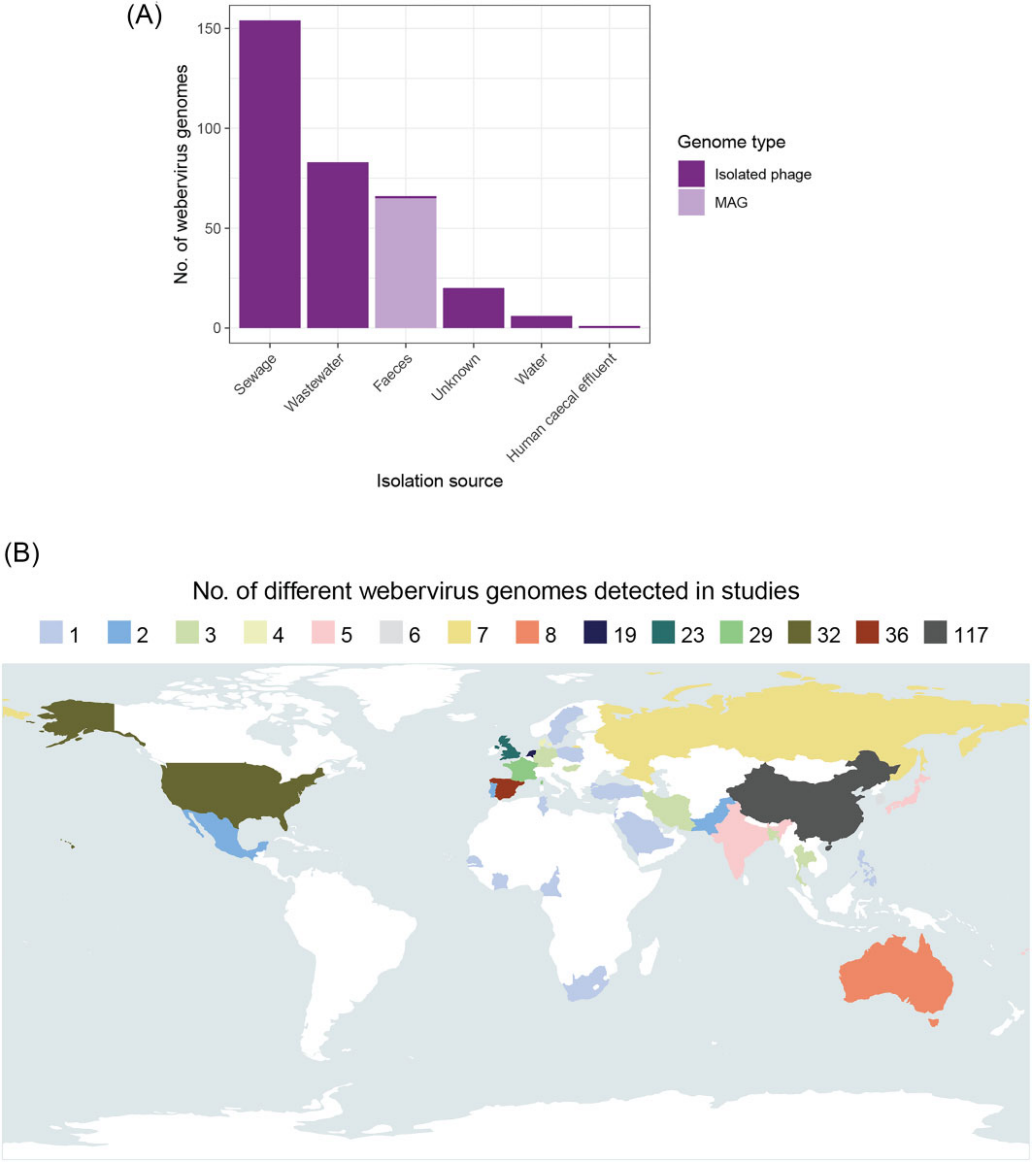
Distribution of weberviruses (A) Stacked bar graph showing the sources of the 330 webervirus genomes (*n* = 265 isolated phages; *n* = 65 MAGs). (B) Geographical distribution of 329 of the webervirus genomes included in this study (the location information was not available for one isolated phage, namely *Klebsiella* phage 5899STDY8049225).

As phages are among the most abundant biological entities on Earth, it is important to gain knowledge on their presence within different environments. We determined that weberviruses are distributed globally and predominated by phages associated with human faeces or water supplies contaminated with human faeces (Fig. 3). Lack of detection in most of South America and Africa is likely due to the absence of metagenomic datasets from these parts of the world rather than weberviruses not being represented in faecal samples from individuals living in countries within these regions. Compared with shotgun metagenomic datasets characterizing the total microbiota found in faeces, there are very few studies—worldwide—examining solely the intestinal virome, and PhageClouds is populated with phage genomes derived from virome datasets.

**Figure 4.**
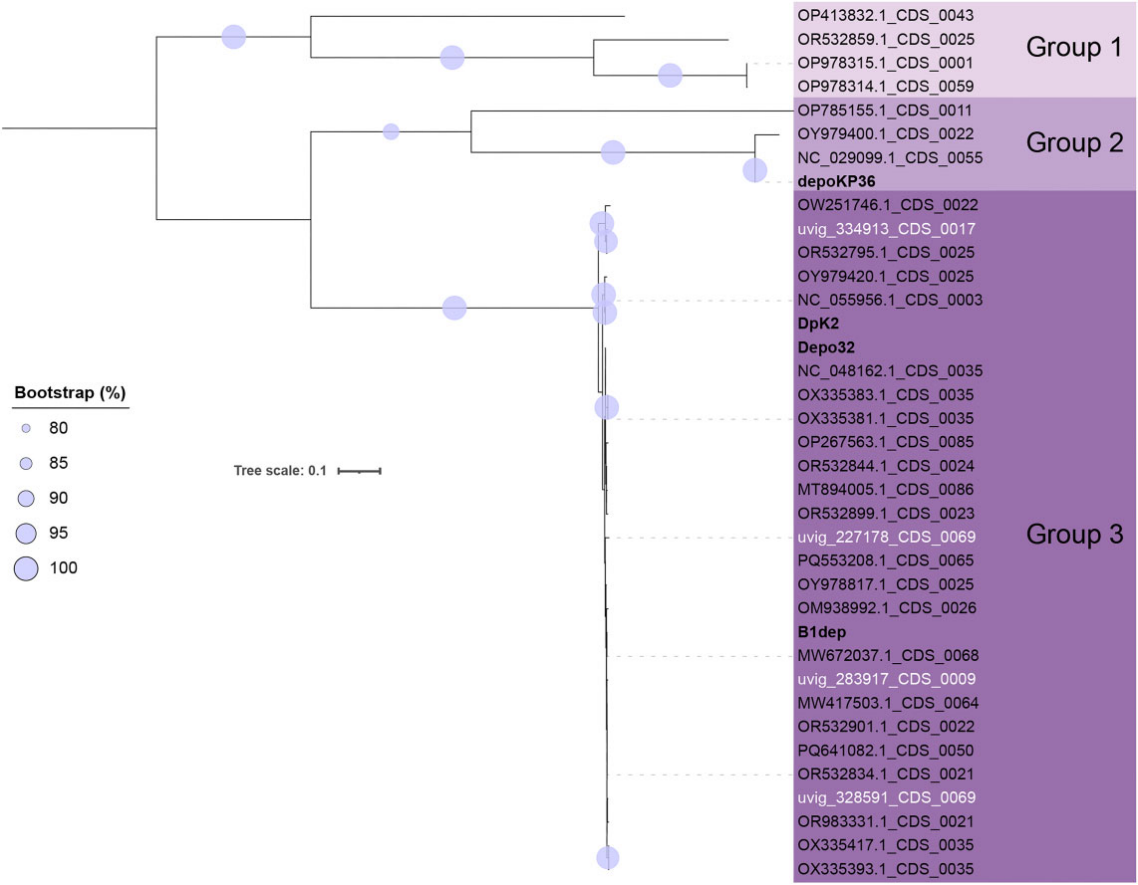
Phylogenetic analysis of depolymerases predicted to be encoded by weberviruses. The tree (maximum likelihood) is rooted at the midpoint. Bootstrap values are presented as a percentage of 100 replicates. Names of experimentally validated (i.e. functional) depolymerases are shown in bold black text; depolymerases predicted to be encoded by MAGs are shown in white text. Scale bar, mean number of amino acid substitutions per position.

It was notable when curating our MAG dataset that none of the studies describing these data were able to predict hosts for the webervirus MAGs we have identified. Nor was the recently released tool iPHoP, specifically designed for use with MAGs (Roux et al. 2023). Our analysis using HostPhinder predicted webervirus MAGs infect *K. pneumoniae*. HostPhinder predicts the host species of a phage by searching for the most genetically similar phages in a database of reference phages with known hosts (Villarroel et al. 2016). Although the authors have shown that this whole-genome similarity-based approach is highly accurate, host range can be altered by a relatively low number of mutations, especially those localized to tail fibre proteins which are often determinants of host-cell specificity (Latka et al. 2021, Taslem Mourosi et al. 2022). In the present study, we used a strain of *K. variicola* to isolate three weberviruses, and phages of this genus have also been isolated on *K. oxytoca* and *K. aerogenes*. Although it is highly likely that 329/330 weberviruses discussed herein are phages of *Klebsiella* spp., determination of host range via plaque assays is still informative, especially when determining therapeutic utility.

## Summary

We successfully characterized seven novel weberviruses that infect clinically relevant MDR *Klebsiella* spp. We have trebled the number of authenticated webervirus genomes through combining genomic data from isolated phage and MAG datasets. In doing so, we have demonstrated the importance of interrogating MAG datasets to expand the availability of curated phage genome sequences for use in genomic and ecological studies, and highlighted the need to exercise caution when assigning low-quality MAGs to taxa.

## Supplementary Material

fiaf043_Supplemental_Files

## Data Availability

The sequences for the seven new phage genomes described herein have been deposited in DDBJ/ENA/GenBank under accession numbers OM065837-OM065843. The genome sequences of bacteria described herein have been deposited under BioProject PRJNA917129. All supplementary material is available from https://figshare.com/projects/Weberviruses_are_gut-associated_phages_that_infect_Klebsiella_spp_/128516.
