# Supplementary material for: Weberviruses are gut-associated phages that infect Klebsiella spp
Source: FEMS Microbiol Ecol. 2025 Apr 18;101(5):fiaf043. doi: 10.1093/femsec/fiaf043 (PMC12023860; doi:10.1093/femsec/fiaf043)
Supplement: fiaf043_Supplemental_Files [file fiaf043_supplemental_files.zip › Supplementary_Figures.docx]

**Supplementary figures for Dawson *et al.*, Weberviruses are gut-associated phages that infect *Klebsiella* spp.**


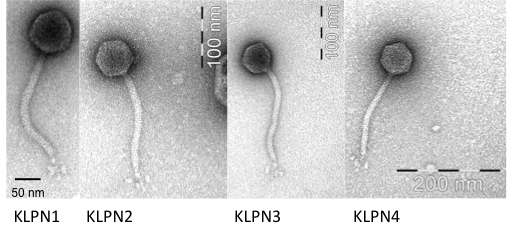


(d)

(c)

(b)

(a)


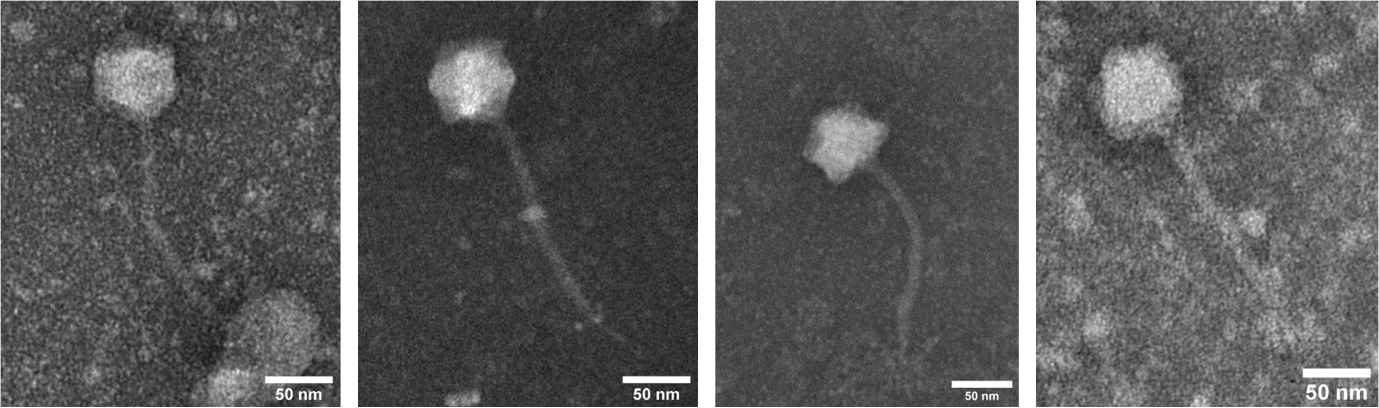


(h)

(g)

(f)

(e)

**Supplementary Figure A.** TEM images of (a) KLPN1, (b) vB_KpnS-KLPN2, (c) vB_KpnS-KLPN3, (d) vB_KpnS-KLPN4, (e) vB_KvaS-KLPN5, (f) vB_KvaS-KLPN6, (g) vB_KvaS-KLPN7 and (h) vB_KpnS-KLPN8.

**
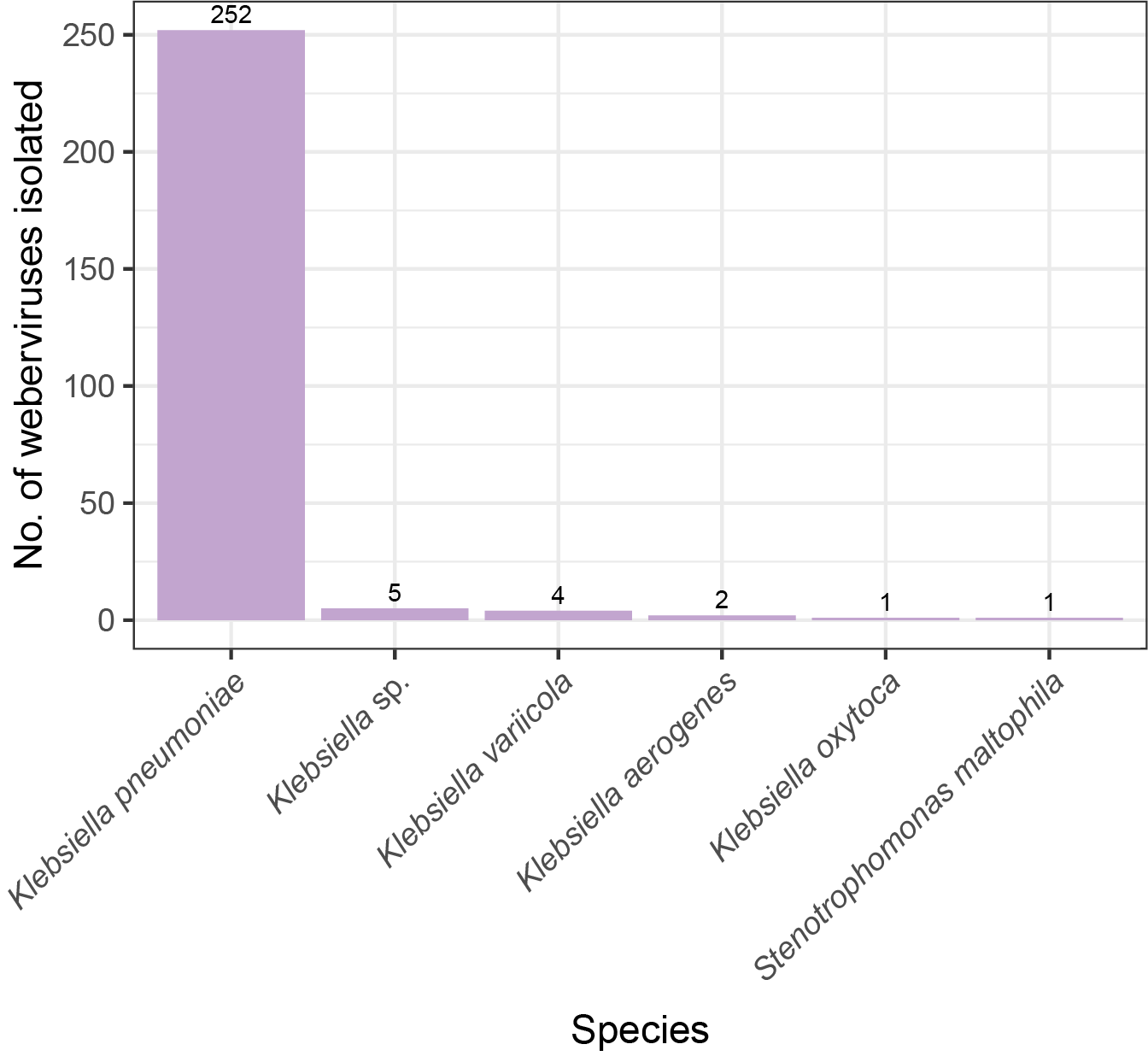
**

**Supplementary Figure B.** Weberviruses are most frequently isolated on *Klebsiella* spp., particularly *K. pneumoniae*. The isolation sources of the 265 isolate genomes (**Supplementary Table 2**) were collated and are summarized here.


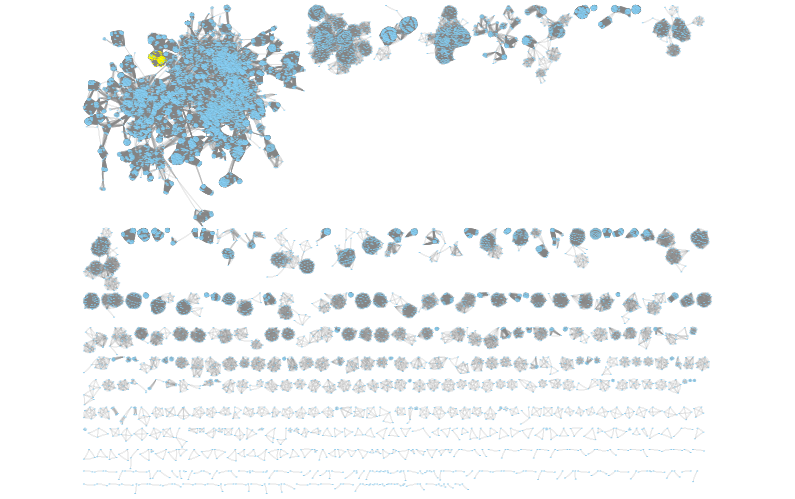


**Supplementary Figure C.** Full gene-based network generated using all genomes listed in **Supplementary Table 2** and **Supplementary Table 3**. Weberviruses are shown in yellow at the top left-hand side of the image. These and their first and second neighbours were identified and used to generate the subnetwork shown in **Figure 2(b)**.

**
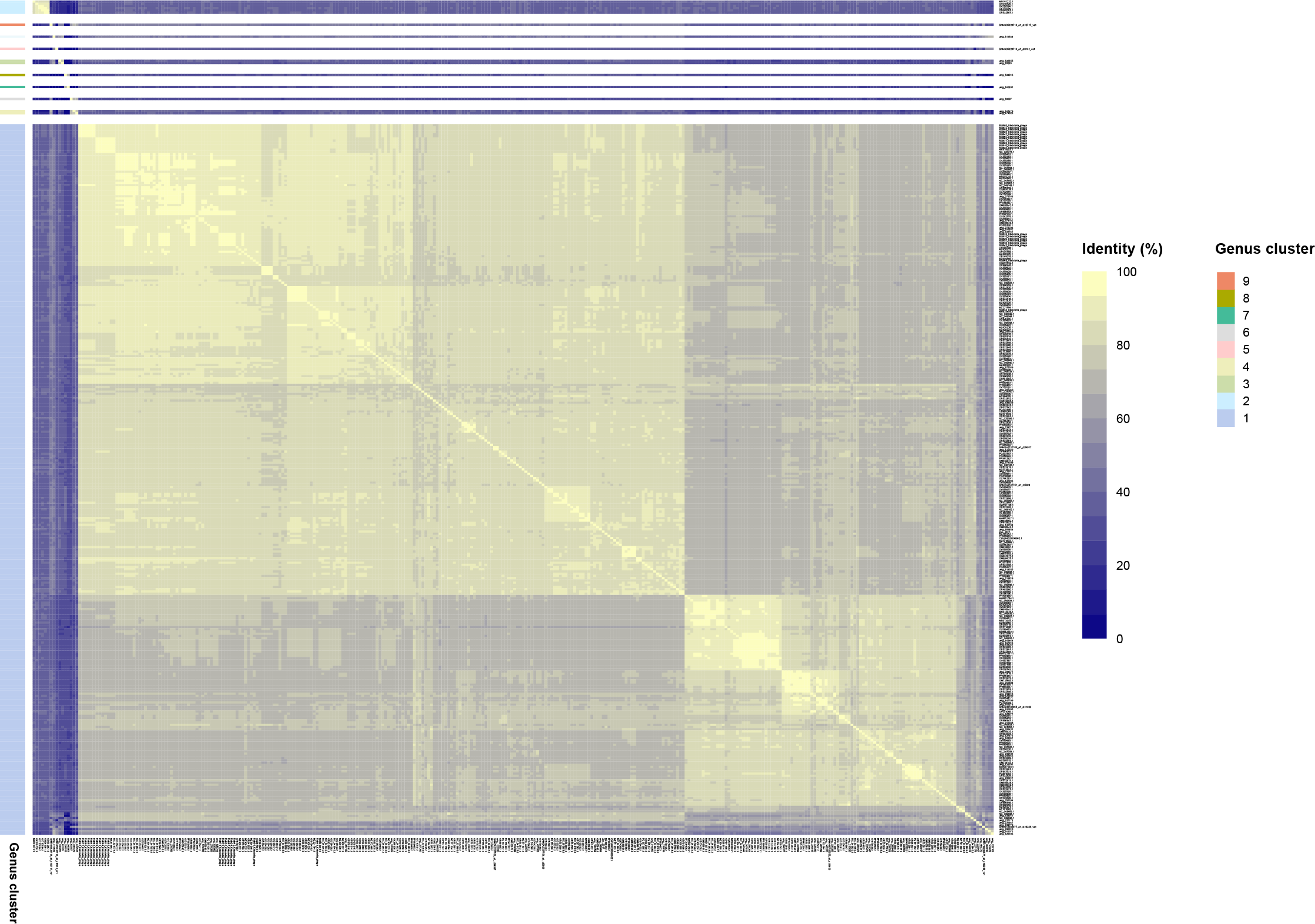
**

**Supplementary Figure D.** Heatmap showing bidirectional clustering of taxmyPHAGE similarity data for the 330 webervirus genomes and those of the novel genus *Defiantjazzvirus*. The numbered clusters correspond to the nine genus clusters detected by taxmyPHAGE with our curated set of sequence data.


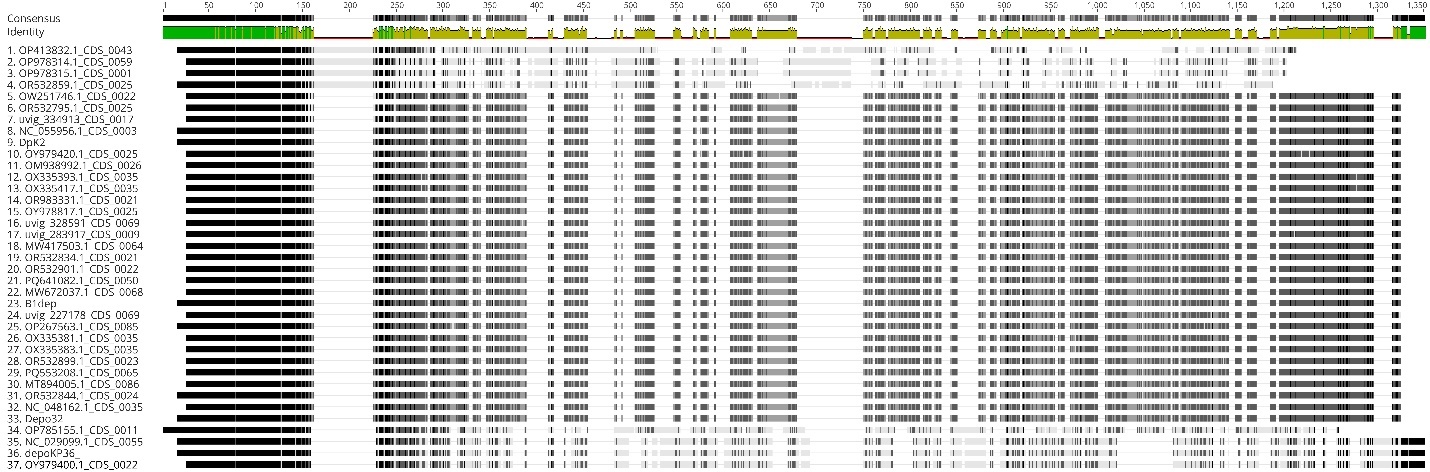


**Supplementary Figure E.** Amino acid alignment (Clustal Omega) of depolymerases identified in this study. A high level of sequence conservation (indicated in green) was identified at the N-terminal region of all the depolymerase sequences (first 100 amino acids).
